# Targeted Cancer Therapy via pH-Functionalized Nanoparticles: A Scoping Review of Methods and Outcomes

**DOI:** 10.3390/gels8040232

**Published:** 2022-04-11

**Authors:** Stefan Morarasu, Bianca Codrina Morarasu, Razvan Ghiarasim, Adina Coroaba, Crina Tiron, Radu Iliescu, Gabriel-Mihail Dimofte

**Affiliations:** 1 Regional Institute of Oncology (IRO), 700483 Iasi, Romania; transcendctiron@iroiasi.ro (C.T.); radu.iliescu@umfiasi.ro (R.I.); gdimofte@gmail.com (G.-M.D.); 2 Internal Medicine Department, Saint Spiridon University Hospital, Grigore T Popa University of Medicine and Pharmacy, 700483 Iasi, Romania; morarasu.bianca.codrina@gmail.com; 3 Centre of Advanced Research in Bionanoconjugates and Biopolymers Department, “Petru Poni” Institute of Macromolecular Chemistry, 700483 Iasi, Romania; ghiarasim.razvan@icmpp.ro (R.G.); adina.coroaba@icmpp.ro (A.C.)

**Keywords:** pH-responsive nanoparticles, drug delivery, cancer therapy, nanocarriers

## Abstract

(1) Background: In recent years, several studies have described various and heterogenous methods to sensitize nanoparticles (NPs) to pH changes; therefore, in this current scoping review, we aimed to map current protocols for pH functionalization of NPs and analyze the outcomes of drug-loaded pH-functionalized NPs (pH-NPs) when delivered in vivo in tumoral tissue. (2) Methods: A systematic search of the PubMed database was performed for all published studies relating to in vivo models of anti-tumor drug delivery via pH-responsive NPs. Data on the type of NPs, the pH sensitization method, the in vivo model, the tumor cell line, the type and name of drug for targeted therapy, the type of in vivo imaging, and the method of delivery and outcomes were extracted in a separate database. (3) Results: One hundred and twenty eligible manuscripts were included. Interestingly, 45.8% of studies (n = 55) used polymers to construct nanoparticles, while others used other types, i.e., mesoporous silica (n = 15), metal (n = 8), lipids (n = 12), etc. The mean acidic pH value used in the current literature is 5.7. When exposed to in vitro acidic environment, without exception, pH-NPs released drugs inversely proportional to the pH value. pH-NPs showed an increase in tumor regression compared to controls, suggesting better targeted drug release. (4) Conclusions: pH-NPs were shown to improve drug delivery and enhance antitumoral effects in various experimental malignant cell lines.

## 1. Introduction

The advancements made in nanotechnology in recent years has led to an unprecedented interest in developing targeted therapies for cancer based on nanoparticles (NPs). NPs are defined as nano-sized particles with diameters ranging from 1 to 100 nm [1,2,3]. Although small, NPs have a large surface area and can be used as carriers for a wide range of peptides [4], antibodies [5], drugs [6], or contrast agents [7]. NPs are widely used as a platform for delivering drugs due to their stable high carrier capacity and their ability to accumulate in tumors through the enhanced permeation and retention effect (EPR) [8,9]. Because of the accelerated angiogenesis, tumors are supplied by immature blood vessels with a defective architecture with wide endothelial gaps through which molecules smaller than 700 nm can penetrate [10,11,12]. This characteristic represents the core which led to NPs becoming an important platform for research into cancer theranostics. Inversely, many tumors are heterogenous and possess a dense extracellular matrix which increases interstitial pressure by blocking the passive transport of NPs from the peritumoral vessels [9], which explains why NPs mostly accumulate in the peritumoral region but fail to penetrate the deep tumoral tissue in experimental applications.

Studies have described techniques to improve the penetration of NPs by using the tumor microenvironment as a targeting site for NPs. One of the constant distinct features of the tumoral microenvironment is the acidic pH, between 0.3 to 0.7 units lower than the pH of normal tissue [13]. Based on this trait, several studies have designed functionalized NPs, making them responsive to pH changes. Once the pH-functionalized NPs (pH-NPs) penetrate through the endothelium via the EPR effect, they respond to the acidic pH and may either disintegrate and release drugs or change their size and shape, thus enhancing their capacity to diffuse towards the tumors’ core. In recent years, several studies have described various and heterogenous methods to sensitize NPs to pH changes; thus, in this current scoping review, we aimed to map current protocols for pH functionalization and analyze the antitumoral outcomes of drug-loaded pH-NPs.

## 2. Materials and Methods

### 2.1. Literature Search and Study Selection

As previously described [14,15,16], a systematic search of the PubMed database was performed for all published studies relating to in vivo models of anti-tumor drug delivery via pH-responsive NPs using the following search algorithm: pH AND nanoparticles AND cancer AND delivery AND in vivo. The systematic search was carried out by adhering to the Preferred Reporting Items for Systematic Reviews and Meta-Analyses (PRISMA) guidelines which were adapted to experimental studies [17]. The PRISMA checklist was followed to conduct the methodology. Inclusion criteria were used according to the Problem/Population, Intervention, Comparison, and Outcome (PICO) formula (Table 1). All studies published in English from the 1st of January 2017 to the 31th of December 2021 describing drug-loaded pH-responsive NPs for targeted delivery in tumors were selected for full-text review. The experimental lot (population) consisted of pH-functionalized nanoparticles tested in vitro to assess pH responsiveness and in vivo to assess the antitumoral effects of pH-NPs loaded with chemotherapeutics. Embryos, cell cultures, tumor spheroids, and human studies were excluded. Nanogels or nano-emulsions were excluded. The intervention was defined as administration of pH-responsive conjugated NPs in tumor-bearing animals. Comparison criteria were further selected from subgroups of the included studies. Primary outcomes were tumor uptake of pH-NPs and tumor regression rate.

### 2.2. Data Analysis

The following data information regarding each included study was extracted: the author name, the year of publication, the type of NPs, the pH sensitization method, the in vivo model, the tumor cell line, the type and name of drug used for targeted therapy, the type of in vivo imaging, method of delivery, and the outcomes regarding the cellular uptake of NPs.

### 2.3. Quality Assessment

**Figure 1:**
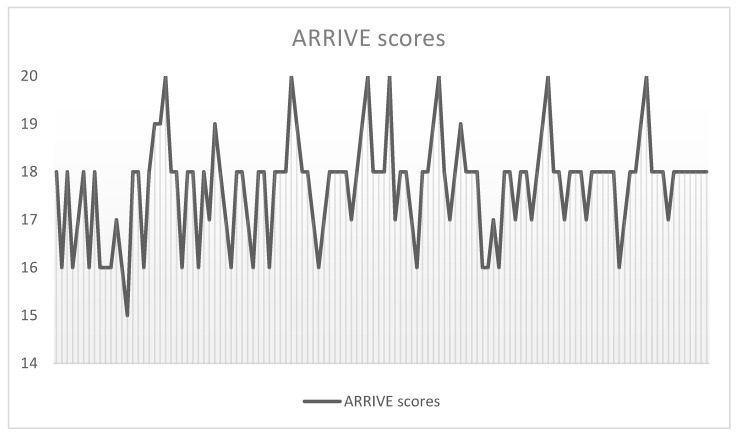
ARRIVE scores breakdown of included studies.

Two authors (SM and BCM) independently examined the title and abstract of citations, and the full texts of potentially eligible studies were obtained; disagreements were resolved by discussion. The Essential 10 ARRIVE guidelines were used to quantify the quality of included studies [18]. Each study was marked for each ARRIVE item with 0 if the data were lacking, 1 if the data were incomplete, and 2 if the data were complete; thus, the final score of each article could range from zero to a maximum of twenty. Only studies with a minimum ARRIVE score of 14 were included (Figure 1). The reference lists of retrieved papers were further screened for additional eligible publications.

## 3. Results

### 3.1. Overview of Included Studies

**Figure 2:**
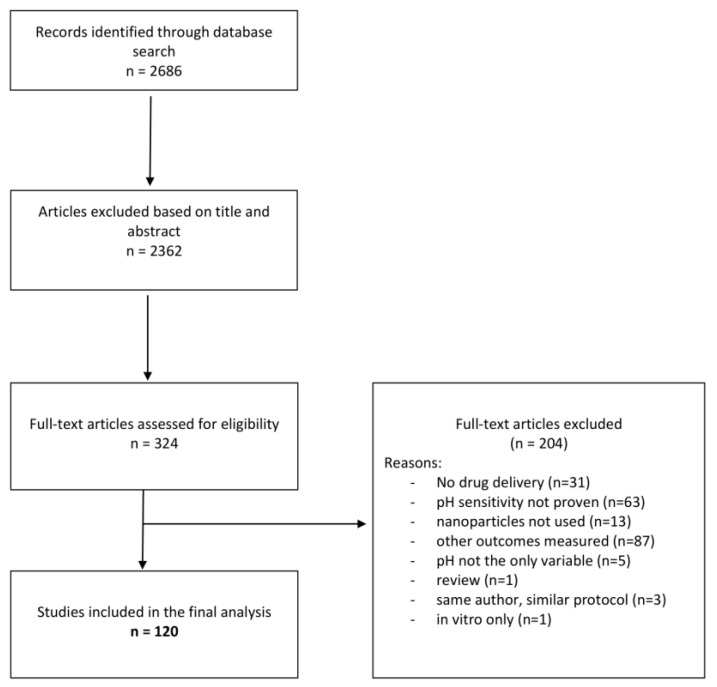
PRISMA flowchart.

An initial search of PubMed database found 2686 articles. After triage of title and abstract, 324 full texts were assessed for inclusion. Records based on the title and abstract were excluded if they did not answer our research question: “Can pH functionalized NPs be used as drug carriers for targeted, in vivo, cancer therapy?”. Further, records were excluded if any of the exclusion criteria were obvious within the title or abstract. Eligible full texts were triaged according to the same principles (Table 1). The PRISMA flowchart shows a breakdown of excluded full texts (Figure 2). One hundred and twenty fully eligible manuscripts were included for in-depth analysis [19,20,21,22,23,24,25,26,27,28,29,30,31,32,33,34,35,36,37,38,39,40,41,42,43,44,45,46,47,48,49,50,51,52,53,54,55,56,57,58,59,60,61,62,63,64,65,66,67,68,69,70,71,72,73,74,75,76,77,78,79,80,81,82,83,84,85,86,87,88,89,90,91,92,93,94,95,96,97,98,99,100,101,102,103,104,105,106,107,108,109,110,111,112,113,114,115,116,117,118,119,120,121,122,123,124,125,126,127,128,129,130,131,132,133,134,135,136,137,138,139] (Table S1). Interestingly, 45.8% of studies (n = 55) used polymers to construct nanoparticles—either natural polymers (such as chitosan) or synthetic ones (Table 2 and Table 3). The most common pH sensitization method used acid-labile bounds (e.g., hydrazone, ester, imide) (Table 2, Table 3, Table 4, Table 5 and Table 6). BALB/c mice were part of the chosen experimental model in 98.3% (n = 118) of studies. pH-NPs were used in a wide array of malignancies, including breast carcinoma (40%, n = 48), hepatocarcinoma (14.1%, n = 17), lung cancer (11.6%, n = 14), colon carcinoma (6.6%, n = 8), cervical cancer (6.6%, n = 8), and melanoma cell lines (1.6%, n = 2) (Table 2, Table 3, Table 4, Table 5 and Table 6). Fluorescent imaging (70.8%, n = 85) and transmission electron microscopy (24.1%, n = 29) were used to quantify in vivo biodistribution of pH-NPs. Most studies (80.8%, n = 97) used control NPs which were not pH-sensitized to compare biodistribution and tumor penetration. Furthermore, almost all researchers (n = 119) compared cargo release from NPs in both physiological and acidic pH. Four studies proved that NPs increase in size when exposed to low pH, due to associated swelling and widening of membrane gaps, before drug release. The mean acidic pH value used in the current literature is 5.7 [5–6.8], which is significantly lower than that measured in tumor microenvironments, which can vary between 6.7 and 7.1, as previously reported.

### 3.2. Types of NPs Used

The sensitization of various NPs to acidic pH was measured. Those that were polymeric in nature were most common (Table 2 and Table 3); however, mesoporous silica nanoparticles (MSNPs) (Table 4), gold-based NPs (Table 5), or lipid-based NPs (Table 6) were other common options. Polymeric NPs were synthetized through emulsion–solvent evaporation methods or by nanoprecipitation. Polymers have the advantage of being biocompatible and biodegradable and can be designed to either incorporate drugs or simply attach drugs to their matrix via pH-labile linkers. Chitosan was commonly used to form nanocomposites because it is a positively charged biocompatible polymer with good stability in blood circulation which can form complexes with anionic peptides. Another way of using polymers in the design of pH-NPs is by coating the surface of other types of NPs to increase in vivo stability (e.g., PEGylated lipid NPs) (Table 6). Polyethylene glycol (PEG) is hydrophilic and biocompatible, thus coating the surface with PEG (e.g., PEGylation) ensured a longer and more stable intravascular circulation with low immunogenicity. MSN-NPs were another widely used platform for designing pH-responsive drug carriers (11.6%, n = 14) synthetized via the solution–gel method (Table 4). Their main advantage is their porous structure which allows inner encapsulation of drugs, but also the surface linkage of tumor-targeting peptides (e.g., folic acid, transferrin) and pH-responsive binders (e.g., imidazole, hydrazine) can prove useful too.

### 3.3. Outcomes of pH-NPs

**Figure 3:**
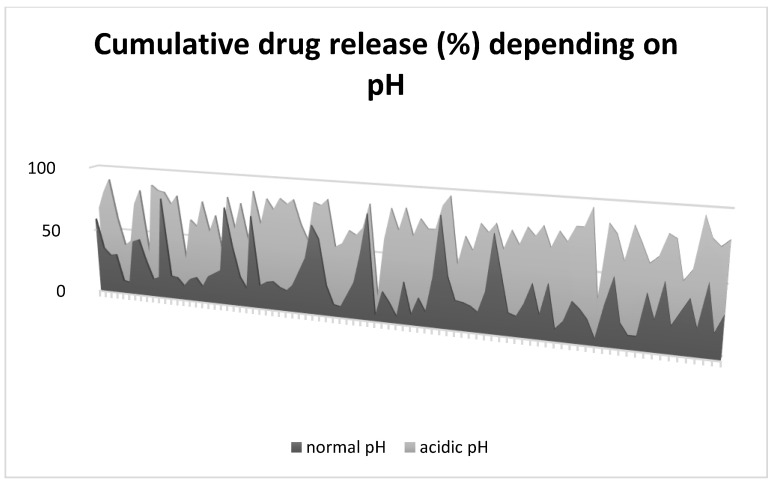
Rate of cumulative drug release for each of the included studies. Dark gray area shows rate (%) of drug released at a physiological pH (7.4). Light gray shows rate (%) of drug released in acidic pH (lowest value used in each study).

**Figure 4:**
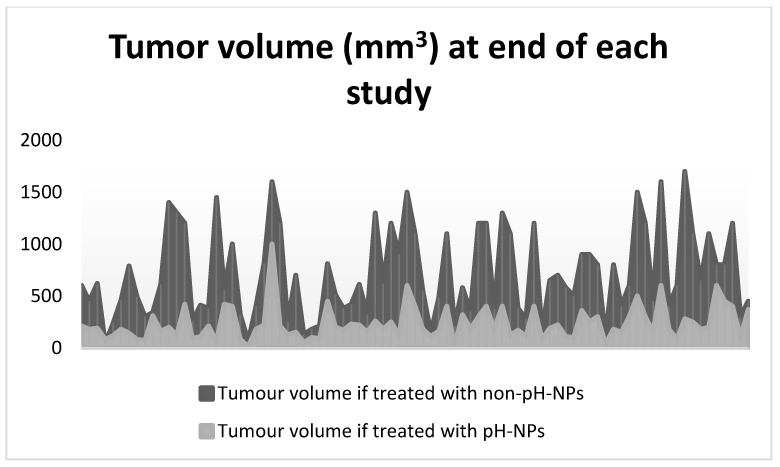
Volume of tumor (mm^3^) at the end of experiment for each of the included studies. Dark gray area shows the tumor volume for specimens treated with non-pH-NPs. Light gray area shows the tumor volume for specimens treated with pH-NPs.

When exposed to in vitro acidic environment, without exception, pH-NPs released drugs inversely proportional to the pH value (Figure 3). In all scenarios, both control and pH-NPs showed similar biodistribution and good stability in vivo; however, pH-NPs showed an increase in tumor regression compared to controls, suggestive of better targeted drug release. As seen in Figure 4, the volume of tumors was lower in groups treated with pH-NPs compared to non-pH-NPs.

## 4. Discussion

Our results show that NPs may be used as pH-responsive platforms with excellent results in tumor penetration and tumor regression rates. pH-NPs, regardless of being metallic or polymeric, were shown to have good tumor penetration in most experimental malignant cell lines in vivo.

Polymers were the most common nanomaterials used in the synthesis of pH-NPs. Besides being used for surface coating to increase the colloidal stability of NPs, polymers (e.g., PEG, PLGA, PHA) were used in the core structure of NPs, making polymeric NPs a widely used platform due to their key advantages: biocompatibility, high stability, non-toxicity, easy synthesis, and versatility. Chemotherapeutics can be linked onto or within the polymers via electrostatic interactions. Once assembled, polymeric NPs have high stability in blood circulation and can maintain the EPR effect, which allows them to escape in the tumoral microenvironment, where drugs are released in a controlled fashion [140]. Mesoporous silica nanoparticles (MSN NPs) were also commonly used to design pH-responsive nanocarriers. The main advantage of MSN NPs is their large surface area and large porous structure, in which a high volume of drugs can be encapsulated. Their surface can be also chemically modified to attach various linkers which react to pH changes [141]. Lipid NPs are usually spherical in shape and formed by a bilayer lipid membrane and an aqueous core. They are highly biocompatible and can transport hydrophilic, hydrophobic, and lipophilic drugs; however, lipid NPs can be cleared by the reticuloendothelial system. For this reason, their surface is usually coated with polymers (e.g., PEGylation) to increase their biostability [142]. Gold NPs can be pH-functionalized using surface pH-responsive linkers. Gold NPs have unique optical characteristics, making them suitable for cancer theranostics and photothermal therapy [143].

The tumor specificity of pH-NPs was further enhanced using tumor-targeting peptides linked to the surface of NPs which can target specific receptors commonly expressed by cancers. The folate receptor is known to be overexpressed in various tumors [144] and was used as a target for NPs coated with folic acid, which facilitates the receptor-mediated endocytosis of NPs, where drug cargo can be released in the acidic intracellular environment. Other studies used Fe ions attached to the surface of NPs, as many tumors use Fe for cellular proliferation [145]. Increased expression of transferrin on tumors promotes NPs attachment and internalization [146]. Xie et al. [120] used methotrexate as an antitumor agent and also as a tumor-targeting agent due to its structural similarity to folic acid and capacity to bind to folate expressed by tumors. Gong et al. [49] used arginine–glycine–aspartate triad (RGD peptide) which is a low-toxicity, highly stable peptide with increased affinity to integrins, which in turn are overexpressed by tumoral neo-vessels.

Doxorubicin is the most used chemotherapeutic in current experiments. Doxorubicin is an anthracycline with potent antimitotic and cytotoxic activity. Its mechanism of action involves intercalation between base pairs where it inhibits DNA synthesis and, in addition, inhibits topoisomerase II activity, thus reducing DNA replication [147,148]. Despite having excellent antitumor activity, its use is limited by important side effects, such as cardiotoxicity and myelosuppression [148]. In a conjugated form, incorporated in the hydrophobic core of nanocarriers, doxorubicin can be administered in higher doses, and can be released at the tumor site where nanoparticles accumulate through enhanced permeability release or by active tumor targeting through pH-dependent conversion, as demonstrated in the included studies.

Drugs are usually loaded into NPs either through core encapsulation or surface bounding. Core encapsulation refers to the organization of NPs around drugs, usually due to their amphipathic property, and the hydrophobic end safeguards the drugs in the center, while the hydrophilic end forms a protective shell, enabling a safe transport of cargo to the tumor. Another way is to attach drugs to the surface of NPs, especially when PEGylation is used to coat the surface. PEG is a stable carrier and binder, and various linkers can be used to attach drugs or tumor-targeting receptors to its surface.

Acid-labile Schiff base linkages were the core from which nanoparticles, regardless of type, were designed to respond to pH changes. Imine Schiff bases undergo hydrolyzation under acidic conditions and such are used as linkers when nanoparticles are assembled. Once the peritumoral acidic pH is sensed, the linkers break, causing disruption of the nanocarriers and release of drugs. In other scenarios, the nanocarriers were coated with tumor-targeting peptides (e.g., folic acid, AS1411 aptamer) which interacted with cancer cells and allowed for the nanocarriers to reach the intracellular environment, via endocytic pathways, where the drugs were released. Another pH sensitization method is the use of electrostatic interactions. pH-NPs were coated with a negative-charged surface which reverted to a positive charge in the acidic environment, leading to the release of positively charged peptides, which were linked to drugs [42].

Functionalized NPs may become a cornerstone in cancer treatment as they can overcome the barrier of systemic toxicity produced by non-targeted chemotherapeutics and can increase the amount of drug delivered to the tumor. Designing NPs responsive to acidic pH has proven to be a solid option. However, we must consider that, in most studies, the maximal effects of pH-NPs were at a pH lower than 6.5. To ensure similar outcomes in clinical studies, pH-NPs need to be ultra-sensitized to release similar amounts of drugs at pH values of 6.8–7.2, which is the usual pH value in the tumor microenvironment.

## 5. Conclusions

This scoping review mapped the current methods and outcomes of using pH-responsive nanoparticles to improve drug delivery and enhance antitumoral effects. Regardless of their type and structure, pH-responsive nanoparticles can increase tumor regression rates compared to the controls. Drug delivery, therefore, is dependent on the exposure of NPs to acidic pH.

## Figures and Tables

**Table 1:** Overview of inclusion and exclusion criteria.

Inclusion Criteria	Exclusion Criteria
Experimental studies	Clinical studies
Full text available in English	Full text not available/other language used
Testing of pH-NPs in vitro and in vivo (animal model)	In vitro/in vivo only
Descriptive data on type and synthesis of NPs	Type of NPs not named/method of synthesis not described
Descriptive data on pH functionalization method	No detailed data on how the NPs were functionalized
Data on animal model and malignant cell line used	No data on animal model/malignant cell line
pH-NPs used to deliver chemotherapeutics	Other use of pH-NPs (e.g., tumor imaging, hyperthermia)
Analysis of tumor uptake of pH-NPs and tumor regression	No data on tumoral response to pH-NPs
Detailed description of methodology (is the method reproducible?)	Methods not reproducible based on given data (requiring supplemental data from authors)
ARRIVE score ≥ 15	ARRIVE score < 15

**Table 2:** Summary of methods used in studies.

	Summary of Studies Overview of Common Methods	
	Type/Method	No. of Studies
**Type of NPs**	Polymeric	55
	Lipid	12
	MSN	13
	Metallic	11
	Other	29
**pH Sensitization Method**	pH-labile linkers	70
	pH-triggered structural changes	35
	pH-triggered hydrophobic to hydrophilic transition	8
	Other methods	7
**Cancer Model**	Breast malignant cell lines (4T1, MCF-7, MDA-MB-231)	48
	Cervical malignant cell lines (HeLA)	8
	Lung malignant cell lines (A549)	14
	Colorectal malignant cell lines (CT-26, HCT116, SW480)	8
	Liver malignant cell lines (H22, HepG2, SMMC 7721)	17
	Other	25
**Types of Chemotherapeutics**	Doxorubicin	69
	Paclitaxel	9
	Other	42

**Table 3:** Overview of polymeric NPs: structure, pH sensitization method, tumor type, and delivered drug.

First Author	Publication Year	Structure of NPs	pH Sensitization Method	Tumor Type	Drug
**Adeyemi** [19]	2019	FA-chitosan-PEG-polyethylenimine	pH-triggered structural changes	KYSE 30 scuamos cell carcinoma	Endostatin
**Cao** [21]	2019	TAT peptide-polyphosphoester	pH sensitive transactivator of transcription (TAT)	MDA-MB-231 breast carcinoma cell line	Doxorubicin
**Chen** [23]	2018	lactobionic acid-chitosan-lipoic acid	pH-labile amide linkers	HepG2 liver cancer	Doxorubicin
**Chen** [25]	2020	TPGS-HA polymer-PEG	hydrophobic to hydrophilic transition	PC3 prostate cancer	Docetaxel
**Cheng** [30]	2019	Poly(ortho ester urethanes) copolymers	pH-labile borate ester linkers	MCF-7 breast carcinoma cell line	Doxorubicin
**Cheng** [28]	2018	carboxymethyl chitosan	pH-labile hydrazone linkers	MCF-7 breast carcinoma cell line	Doxorubicin
**Cui** [31]	2017	transferrin-PEG	pH-labile hydrazone linkers	MCF-7 breast carcinoma cell line	Doxorubicin
**Debele** [32]	2017	PEG-methacrylamide-tocopheryl succinate-histidine	pH-labile imidazole linkers	HCT116 colon carcinoma	Doxorubicin
**Deng** [33]	2019	PEG-methylpropenoic acid-glycerol-cinnamaldehyde	pH-labile cinnamylaldehyde linkers	4T1 breast carcinoma cell line	Doxorubicin
**Du** [36]	2017	PEG-PTTMA	PTTMA disassembly in acidic pH	HeLa cervival cancer	siRNA
**Fan** [39]	2017	polyethylenimine-PEG	pH-labile borate ester linkers	4T1 breast carcinoma cell line	siRNA
**Fang** [40]	2020	chitosan-polysaccharide	pH-labile borate ester linkers	PANC-1 pancreatic cancer	Curcumin
**Feng** [41]	2020	PEG-PAH-DMA	pH-triggered structural changes	A549 NSLC cell line	Paclitaxel
**Gao** [44]	2017	poly (L-γ-glutamylcarbocistein-RBC membrane	pH-triggered structural changes	NCI-H460 cell line	Paclitaxel
**Gibbens-Bandala** [45]	2019	PLGA-polyvinyl alcohol	hydrophobic to hydrophilic transition	MDA-MB-231 breast carcinoma cell line	Paclitaxel
**Gong** [47]	2018	PEG-PPMT	hydrophobic to hydrophilic transition	CT-26 colon carcinoma	Docetaxel
**Guo** [49]	2018	PBLG-Sericin	pH-labile carboxyl linkers	A549 NSLC cell line	Methotrexate
**Guo** [51]	2020	DMA-PEG	pH-triggered structural changes	MCF-7 breast carcinoma cell line	Doxorubicin
**Hong** [53]	2019	U11 peptide-PLGA	pH-triggered structural changes	A549 NSLC cell line	Doxorubicin and Curcumin
**Jin** [57]	2018	PEI-PLA	pH-triggered structural changes	A549 NSLC cell line	Paclitaxel
**Jung** [58]	2020	PBA	pH-labile borate ester linkers	MG glioblastoma	Doxorubicin
**Khan** [61]	2020	PLGA	pH-triggered structural changes	MCF-7 breast carcinoma cell line	Doxorubicin
**Kou** [64]	2017	lactose myristoyl carboxymethyl chitosan	pH-triggered structural changes	Huh-7 hepatocellular carcinoma	Adriamycin
**Lee** [66]	2018	chitosan-PEG-acetyl histidine	pH-triggered structural changes	CT-26 Pulmonary Metastasis Model	Piperlongumine
**Li** [70]	2018	DGL-PEG-Tat-KK-DMA	pH-labile amide linkers	HepG2 liver cancer	Doxorubicin
**Li** [73]	2020	RGD-PEG-Arginine-SA	pH-labile hydrazone linkers	HN6 squamos cell carcinoma	GNA002
**Li** [75]	2021	PDA-HA	pH-labile PDA coating	4T1 breast carcinoma cell line	Cisplatin
**Liu** [79]	2018	polycarbonate-PEG	pH-labile acetal linkers	BT 474 breast carcinoma	Bortezomib
**Luo** [87]	2021	PEG-TAT-HA	pH-triggered structural changes	H22 hepatocellular carcinoma	Disulfiram
**Mhatre** [89]	2021	polydopamine	pH-triggered structural changes	MDA-MB-231 breast carcinoma cell line	Niclosamide
**Palanikumar** [96]	2020	ATRAM-BSA-PLGA	pH-labile ester bonds	MCF-7 breast carcinoma cell line	Doxorubicin
**Qu** [100]	2018	carboxymethyl chitosan	pH-labile phenylboronic acid pinacol ester	HepG2 liver cancer	Doxorubicin
**Quadir** [101]	2017	PEG-PPLG	pH-labile amine linkers	MCF-7 breast carcinoma cell line	Doxorubicin
**Ray** [102]	2020	PEG	pH-labile amine linkers	PANC-1 pancreatic cancer	Gemcitabine
**Saravankumar** [103]	2019	APT-PLGA-PVP-AS1411 aptamet	pH-triggered structural changes	A549 NSLC cell line	Doxorubicin
**Shi** [105]	2018	PEG-PLH	pH-labile PSD linker	A549 NSLC cell line	siRNA
**Shi** [106]	2021	PEG-PLL-DMA	pH-labile amide linkers	A549 NSLC cell line	siRNA
**Soe** [107]	2019	poloxamer-Tf-EDC-NHS	NR	MDA-MB-231 breast carcinoma cell line	Doxorubicin
**Su** [108]	2020	PEG-PMT	pH-labile tioether linkers	Colon26 cell line	Docetaxel
**Wang** [113]	2017	RGD-PLGA-PEG	pH-labile amine linkers	MCF-7 breast carcinoma cell line	Doxorubicin
**Wang** [115]	2018	chitosan-graphene oxide	pH-triggered structural changes (less electrostatic interaction	HepG2 liver cancer	Doxorubicin
**Wei** [118]	2020	PEG	pH-labile amine linkers (schiff base)	B16F10 melanoma	Doxorubicin
**Xiong** [122]	2019	TPGS-PEG	pH-labile hydrazone linkers	MCF-7 breast carcinoma cell line	Doxorubicin
**Xu** [123]	2018	DTPA-PEG-DMA	pH labine amine linkers	PC3 prostate cancer	Doxorubicin
**Xu** [124]	2021	chitosan	pH-labile ester linkers	HepG2 liver cancer	Doxorubicin
**Yadav** [125]	2020	RGD-chitosan-Cy5.5	pH-labile amine linkers	MDA-MB-231 breast carcinoma cell line	Raloxifene
**Yan** [126]	2017	POEAd-galactose-LA	pH-labile ester linkers	HepG2 liver cancer	Doxorubicin
**Yang** [127]	2018	glycol Chitosan-PDPA	hydrophobic to hydrophilic transition (PDPA)	MCF-7 breast carcinoma cell line	Paclitaxel
**Yu** [128]	2019	PLGA-CPT-DMMA-PEI	pH-triggered structural changes	MCF-7 breast carcinoma cell line	Doxorubicin
**Zhang** [129]	2017	TPGS-MSN	pH-labile ester linkers	SMMC 7721 hepatocellular carcinoma	Doxorubicin
**Zhang** [131]	2018	DMA-Cystamine-PEG	pH-labile ester linkers	A549 NSLC cell line	Paclitaxel
**Zhou** [138]	2020	polyphosphazene	pH-labile hydrazone linkers	HeLa cervival cancer	Doxorubicin

Legend: FA, folic acid; TPGS, tocopheryl polyethylene glycol 1000 succinate; HA, hyaluronic acid; PEG, polyethylene glycol; PTTMA, poly(2,4,6-trimethoxybenzylidene-1,1,1-tris(hydroxymethyl)ethane methacrylate; DMA, dimethylmaleic acid; PAH, polyallylamine; RBC, red blood cell; PLGA, poly(lactic-co-glycolic acid); PPMT, poly(o-pentadecalactone-co-N-methyldiethyleneamineco-3,30-thiodipropionate; PBLG, poly(c-benzyl-L-glutamate); U11 peptide, urokinase plasminogen activator receptor (uPAR) targeting peptide; PEI, polyethyleneimine; PLA, polylactic acid; PBA, phenylboronic acid; DGL, dendrigraft poly-L-lysine; TAT, tumor-associated antigens; RGD, arginine–glycine–aspartic peptide; DTPA, 3,3′-dithiodipropionic acid; Cy5.5, cyanine; SA, stearic acid; PDA, hydrochloride dopamine; ATRAM, acidity-triggered rational membrane peptide; BSA, bovine serum albumin; PPLG, poly (γ-propargyl L-glutamate); APT, aptamer; PVP, poly(N-vinylpyrrolidone); PLH, poly(L-histidine); PLL, poly-L-lysine; EDC, 1-ethyl-3-(3-dimethylaminopropyl)-carbodiimide hydrochloride; NHS, N-hydroxysuccinimide; PMT, poly(ω-pentadecalactone-co-N-methyldiethyleneaminesebacate-co-2,2’-thiodiethylene sebacate); DTPA, 3,3′-dithiodipropionic acid; POEAd, poly(ortho ester diamide); LA, lactobionic acid; PDPA, poly(2-(diisopropylamino)ethyl methacrylate); CPT, C18-PEG2000-TPP.

**Table 4:** Overview of mesoporous silica NPs: structure, pH sensitization method, tumor type, and delivered drug.

First Author	Publication Year	Structure of NPs	pH Sensitization Method	Tumor Type	Drug
**Chen** [24]	2020	MSN-citraconic-poly-L-lisine	acid-labile disulfide linkers	4T1 breast carcinoma cell line	Doxorubicin
**Cheng** [27]	2017	Polydopamine-FA-PEG-MSN	pH-labile polydopamine coating	HeLa cervival cancer	Doxorubicin
**Ding** [34]	2020	MSN-carboxymethyl chitin-GRP78 peptide	pH-labile thioketal linkers	H22 hepatocellular carcinoma	Doxorubicin
**Ding** [35]	2020	MSN-lipidbilayer-TLS11a aptamer	pH-labile TAT peptide	4T1 breast carcinoma cell line	Doxorubicin
**Kundu** [65]	2020	MSN-FA	pH-labile PAA linker	MCF-7 breast carcinoma cell line	Umbelliferone
**Li** [73]	2020	Gal-P123-MSN	pH-triggered structural changes (DC lipid)	Huh-7 hepatocellular carcinoma	Irinotecan
**Li** [68]	2017	DM1-MSN-PDA	pH-labile PDA coating	SW480 colorectal cancer cell line	EpCAM
**Liao** [76]	2021	Chitosan-MSN	pH-labile imidazole linkers	4T1 breast carcinoma cell line	Doxorubicin
**Liu** [80]	2019	MSN	pH-labile calcium carbonate	LNCaP-AI prostate carcinoma	Doxorubicin
**Mu** [94]	2017	MSN-PLH-PEG	hydrophobic to hydrophilic transition	H22 hepatocellular carcinoma	Sorafenib
**Saroj** [104]	2018	MSN	pH-labile PAA linker	PC3 prostate cancer	Bicalutamide
**Zhang** [130]	2017	MSN-pH-responsive peptide	pH-responsive peptide	MCF-7 breast carcinoma cell line	Doxorubicin
**Zhao** [136]	2018	MSN-TPGS	pH-labile ester linkers	MCF-7 breast carcinoma cell line	Doxorubicin

Legend: MSN, mesoporous silica nanoparticles; FA, folic acid; PEG, polyethylene glycol; GRP78P, glucose regulated protein 78 peptide; TAT, tumor-associated antigens; Gal, gala tosyl; DM1, maytansinoid conjugate; PDA, hydrochloride dopamine; PLH, D-alpha-tocopherol polyethylene glycol 1000-succinate; PAA, polyacrylic acid.

**Table 5:** Overview of gold NPs: structure, pH sensitization method, tumor type, and delivered drug.

First Author	Publication Year	Structure of NPs	pH Sensitization Method	Tumor Type	Drug
**Aguilar** [20]	2021	polycaffeic acid-FA-Au	pH-labile catechol-boronic acid linkers	SCC7 squamos cell carcinoma	Bortezomib
**Essawy** [38]	2020	Au-hydrazine	pH-labile hydrazone linkers	HBPC oral carcinoma	Doxorubicin
**Guo** [50]	2018	Au-Chitosan-AS1411 aptamer	pH-triggered structural changes	A549 lung cancer cell line	Methorexate
**Kumar** [63]	2020	Au	pH-labile peptide linker (Lys-Phe-Gly)	BT 474 breast carcinoma	Doxorubicin
**Liu** [81]	2018	Au-iron oxide-PEG	pH-labile oleic acid linkers	SGC-7901 gastric adenocarcinoma	Herceptin
**Mahalunkar** [91]	2019	Au-PVP-FA	pH-triggered structural changes	MCF-7 breast carcinoma cell line	Curcumin
**Sun** [110]	2019	Au-AS1411 aptamer	pH-triggered structural changes	HeLa cervival cancer	Doxorubicin

Legend: FA, folic acid; Au, gold; PEG, polyethylene glycol; PVP, polyvinylpyrrolidone.

**Table 6:** Overview of lipid-based NPs: structure, pH sensitization method, tumor type, and delivered drug.

First Author	Publication Year	Structure of NPs	pH Sensitization Method	Tumor Type	Drug
**Juang** [59]	2019	lipid-PEG	pH-labile imide linkers	HCT116 colon carcinoma	Irinotecan and microRNA
**Li** [69]	2017	TF-PEG-GMS	pH-labile hydrazone linkers	A549/DTX lung cancer cell line	Docetaxel and Baicalin
**Li** [71]	2019	LDL-OA	pH-labile hydrazone linkers	4T1 breast carcinoma cell line	Doxorubicin
**Sun** [111]	2021	DSPE-PEG	pH-triggered structural changes	LNCaP-AI prostate carcinoma	Doxorubicin
**Tan** [112]	2017	PAA-OA	pH-labile oleic acid linkers	A549 NSLC cell line	Erlotinib
**Men** [92]	2020	lipid-HA-PBAE	pH-triggered structural changes	A549 NSLC cell line	Doxorubicin
**Cavalcante** [22]	2021	DSPE-PEG-OA	pH-labile oleic acid linkers	4T1 breast carcinoma cell line	Doxorubicin
**Li** [67]	2017	DSPE-PEG	pH-labile imine linkers	FTC-133 thyroid cancer	Doxorubicin
**Lo** [85]	2020	DSPE-PEG	pH-labile oleic acid linkers	SAS squamos carcinoma cell line	Daunorubicin and Irinotecan
**Ma** [90]	2021	DSPE-PEG	pH-triggered structural changes	HepG2 liver cancer	hydroxycamptothecin
**Pang** [98]	2020	lipid-polymeric NPs	pH-labile dihydrazide linkers	A549 NSLC cell line	Erlotinib
**Xie** [120]	2018	DSPE-PEG	pH-labile imine linkers	MCF-7 breast carcinoma cell line	Methotrexate

Legend: PEG, polyethylene glycol; TF; transferrin; GMS, glyceryl monostearate; PAA, polyacrylic acid; HA, hyaluronic acid; PBAE, poly(b-amino ester; DSPE, 1,2-distearoyl-sn-glycero-3-phosphoethanolamine; OA, oleic acid.

## Data Availability

Data can be made available at request.

## Supplementary Materials

The following supporting information can be downloaded at: https://www.mdpi.com/article/10.3390/gels8040232/s1, Table S1: Detailed Overview of Included Studies.
